# The association of sex and attitudes towards parental migration with anxiety symptoms in left-behind children: a large-scale cross-sectional study

**DOI:** 10.3389/fpsyt.2025.1716070

**Published:** 2026-01-12

**Authors:** Jizhou Liu, Chaodang Zhou, Hongming Liang, Yonglan Yang, Yanping Li, Mei He, Ning Zhou, Jinfeng Li

**Affiliations:** 1 Department of Psychiatry, Hospital for Infectious Diseases, Honghe Hani and Yi Autonomous Prefecture, Mengzi, Yunnan, China; 2 Department of Clinical Psychology, The Second People’s Hospital of Honghe Hani and Yi Autonomous Prefecture, Jianshui, Yunnan, China; 3 Department of Pharmacy, The Fifth Affiliated Hospital of Zhengzhou University, Zhengzhou, China

**Keywords:** anxiety, attitude, left-behind children, mental health, parental migration

## Abstract

**Background:**

Left-behind children (LBC) in China, especially in ethnic minority regions, face unique psychosocial challenges due to parental migration, with anxiety symptoms (AS) among the most common mental health issues. This study examined the prevalence of AS in LBC, explored associated socio-demographic and left-behind characteristics, and identified independent risk factors.

**Methods:**

A cross-sectional study was conducted among 856 students aged 7–17 years from three counties in southern Yunnan Province, with 829 included in the final analysis. Socio-demographic and left-behind information was collected via self-administered questionnaires. AS were assessed using the Screen for Child Anxiety-Related Emotional Disorders (SCARED). Group comparisons used t-tests, Mann–Whitney U tests, and chi-square tests. Logistic regression identified independent risk factors, and multivariate linear regression examined associations with SCARED scores.

**Results:**

AS prevalence was higher in LBC than non-LBC (44.21% vs. 22.75%; χ² = 28.170, P < 0.001; OR = 2.285, 95% CI: 1.658-3.149). Among LBC, females and children with neutral or opposed attitudes toward parental migration were more likely to report AS. Logistic regression confirmed female sex (B = 0.663, OR = 1.940, 95%CI=1.377-2.732) and neutral attitude toward migration (B = 0.740, OR = 2.096, 95%CI=1.338-3.283) as independent risk factors. Higher SCARED scores were additionally associated with less frequent communication with migrant parents (B = 1.996), neutral/opposed attitudes (B = 1.880), and lack of a help-seeking figure (B = 3.020).

**Conclusion:**

LBC have a 2.285-fold higher AS prevalence than non-LBC. Female sex, neutral/opposed attitudes toward parental migration, reduced parental communication, and insufficient social support exacerbate anxiety severity, highlighting the need for targeted interventions.

## Highlights

- This is the first study estimating the association of sex and attitudes towards parental migration with anxiety symptoms (AS) in left-behind children (LBC).
- The prevalence of AS was 2.285 times higher in the LBC group (44.21%) than in the non-LBC group (22.75%), suggesting the need to care for the mental health of children left behind.
- “Sex-Female”, “Attitude-Neutrality”, and “Attitude-Opposition” showed significant associations with AS, indicating female and those who are “Attitude-Neutrality” or “Attitude-Opposition” deserve special attention among the children left behind.
- These findings support some new management options such as introducing a care policy for LBC, especially for female in LBC and guidance for parents of LBC to communicate effectively with their children for the purpose of adjusting the attitudes of LBC towards their parents’ migration.

## 1 Introduction

Left-behind children (LBC) are those under 18 who remain at home while one or both parents migrate for work for at least six consecutive months. A systematic review and meta-analysis of low- and middle-income countries found that the prevalence of LBC is significant in several regions, such as 27% in the Philippines, 36% in Ecuador, and over 40% in rural South Africa (1). In China, the largest middle-income country over the past 40 years, many Chinese parents have relocated from rural to urban areas in search of higher-paying jobs to boost family income, leading to a large population of LBC in rural regions, which may face considerable mental health challenges. A study found that more than 68 million children were left-behind by one or both migrating parents, representing 25% of the national child population (2).

Considerable attention has been paid to the mental health of LBC. Anxiety, the most common mental health condition among children and adolescents, is linked to increased risks of suicidal thoughts (3). Furthermore, anxiety has a prevalence rate of 13.2%–57.6% in LBC (4) compared to 1.5%-21% in children and adolescents globally (3). Consequently, we should pay attention to the anxiety symptoms (AS) of LBC. However, conflicting evidence exists regarding the mental health status of LBC. On the one hand, studies have shown that LBC experience higher levels of anxiety than children who are not left behind. For example, a study found that LBC exhibited elevated anxiety levels compared to non-LBC (5). Additionally, some researchers found higher anxiety levels in LBC compared to children living with both parents (6). On the other hand, however, some studies have shown that being left behind does not directly correlate with mental health problems in children, highlighting the complexity of this issue. For instance, some studies found no significant differences in mental health between LBC and those who were not left behind (7). Additionally, a cross-sectional study in China indicated that parental migration was not associated with anxiety in children (8). Furthermore, a study in Ethiopia found no significant effect of parental migration on children (9). Therefore, the association between being left behind and AS remains unclear and controversial, warranting further investigation.

Numerous studies have shown that females are more susceptible to anxiety, with women being twice as likely to experience anxiety compared to men (10). In a longitudinal analysis, female students exhibited significantly higher anxiety levels than their male counterparts, with a considerably higher percentage of female students surpassing the average anxiety threshold (11). However, research results are inconsistent. For example, a study of patients with fibromyalgia indicated that male may be a risk factor for anxiety (12). The inconsistency of findings suggests that sex differences in AS need further exploration, particularly in special populations such as LBC.

To date, there has been limited research on how LBC’s attitudes toward their migrant parents may influence their anxiety levels. Parental migration can have varying effects on children. On the one hand, working parents can increase family income and improve their standards, which are linked to better family cohesion, trust, and support (13). A study with a sample of 1,350 children found that better family income was positively associated with better health (14). From this perspective, children may be supportive of parental migration. On the other hand, other research has shown that children with migrant parents may face higher risks for issues such as polyvictimization, depression, anxiety, suicidal ideation, stunting, and wasting (1, 15). From this perspective, LBC may not support working parents and may have neutral or opposing views. However, there is limited research on the relationship between LBC’s attitudes toward parental migration and mental health, especially regarding AS. This gap in research is significant, as understanding children’s attitudes may help design more effective interventions. Therefore, this study suggests that attitudes toward parental migration are associated with AS among LBC. This could enable schools and parents to implement appropriate health promotion activities based on children’s views and develop tailored intervention plans.

Accordingly, our current research aims to examined (1) the occurrence of AS in LBC, and (2) the correlation between sex and attitudes towards parental migration with AS in this population.

## 2 Materials and methods

### 2.1 Participants

Ethical approval was obtained retrospectively in 2024 for the secondary analysis of data collected between 2016 and 2017. The study was approved by the Ethics Committee of the Hospital for Infectious Diseases, Honghe Hani and Yi Autonomous Prefecture, Mengzi, Yunnan province, China (ethic approval code: ZSY-2024-003-XJ). Informed consent was obtained from all participants and their guardians before their involvement.

**Figure 1:**
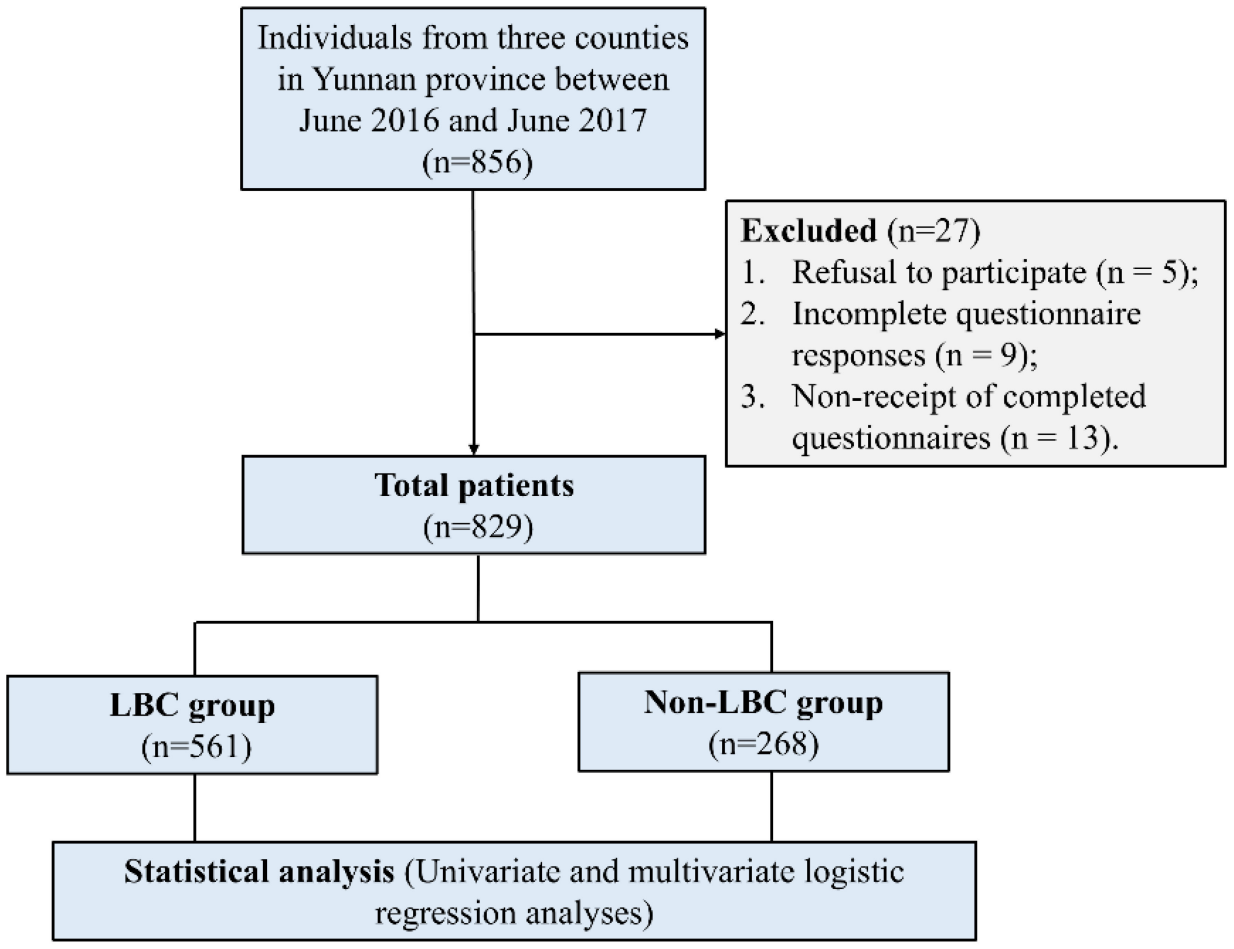
Flowchart of the study. LBC, Left-Behind Children.

A total of 856 individuals from three counties were recruited for this cross-sectional examination conducted between June 2016 and June 2017. The inclusion criteria were as follows (1): current students aged between 7 and 17 years, (2) no diagnosed mental illnesses according to the 5th edition of the Diagnostic and Statistical Manual of Mental Disorders (DSM-V) (16), and (3) residing in areas predominantly occupied by ethnic minorities in China. The exclusion criteria were as follows: (1) individuals with mental illnesses, (2) substance abuse or dependency (excluding tobacco), (3) neurological disorders, and (4) comorbid conditions with other primary Axis I disorders. However, 27 participants were excluded from the analysis due to the following reasons: (1) refusal to participate (n = 5), (2) incomplete questionnaire responses (n = 9), and (3) non-receipt of completed questionnaires (n = 13). Therefore, the final sample size for this study was 829 (**Figure 1**).

### 2.2 Socio-demographic, left-behind features, and AS measurements

Socio-demographic characteristics, such as age, sex, and nation, were collected via self-designed questionnaires. To investigate the left-behind features, the following data were collected: “Only child” (“Yes” or “No”), “Parental migration” (“One parent” or “Both parents”), “Duration of parental migration” (“Less than one year” or “One to two years” or “More than two years”), “Frequency of communication with migrant parent(s)” (“Weekly” or “Monthly” or “More than one month”), “Do you have a favorite animal?” (“Yes” or “No”), “Do you have a favorite plant?” (“Yes” or “No”), “Do you have somebody to talk to?” (“Yes” or “No”), “Do you have someone to ask for help?” (“Yes” or “No”) and “Attitudes towards migration of parent(s)” (“Support” or “Neutrality” or “Opposition”).

To assess AS, the Screen for Child Anxiety-Related Emotional Disorders (SCARED), a widely used screening tool, was employed for children and adolescents based on the DSM criteria. This instrument consists of 41 items that measure overall anxiety, with five factors or subscales specifically targeting different anxiety disorders. These subscales are: F1(Panic/Somatic Symptoms), F2(Generalized Anxiety), F3 (Separation Anxiety), F4 (Social Anxiety), F5 (School Phobia) (17). Additionally, the SCARED tool includes a cutoff score to identify individuals with clinically significant levels of AS, with a score of ≥25 indicating a clinically meaningful level of anxiety (18). The SCARED has demonstrated strong reliability and discriminant validity in various studies. It has also been validated across multiple countries.

### 2.3 Statistical analysis

Statistical analysis was performed using SPSS software (version 26.0; SPSS, Inc., Chicago, IL, USA). The Kolmogorov-Smirnov one-sample test was used to assess the normality of continuous variables. For continuous variables that were normally distributed, independent sample t-tests were conducted to compare the two groups (LBC vs. non-LBC) on the SCARED scores. For continuous variables that were not normally distributed, the Mann-Whitney U test was applied. Categorical variables were compared using the chi-square (χ²) test. Where appropriate, the Bonferroni correction was applied to adjust for multiple comparisons, setting the significance threshold at P < 0.017 (i.e., 0.05 divided by the number of comparisons).

Univariate logistic regression was performed within the LBC group to explore the associations between socio-demographic characteristics, left-behind features, and AS. Variables that met the predefined Bonferroni-adjusted significance threshold in univariate logistic regression analysis were then included in multivariable logistic regression using the Backward: Wald method to identify independent risk factors for AS in LBC children. Additionally, multivariable linear regression analysis was conducted to examine the relationship between the total SCARED score and various clinical factors.

For all statistical tests, a two-tailed significance level was used. Bonferroni correction was applied to baseline comparisons, univariate and multivariable regression analyses to control for multiple testing, and the adjusted significance threshold was set at P < 0.017 for individual comparisons.

## 3 Results

### 3.1 The prevalence of AS in the LBC group compared to the non-LBC group

**Table 1** shows that children in the LBC group scored higher on the SCARED assessment, including the total score, panic/somatic symptoms, generalized anxiety, separation anxiety disorder, social anxiety disorder, and school phobia (P < 0.001), than children in the non-LBC group. Additionally, the prevalence of AS was significantly higher in the LBC group (44.21%) than in the non-LBC group (22.75%) (χ^2^ = 28.170, P < 0.001, OR = 2.285, 95% CI: 1.658-3.149).

**Table 1:** Socio-demographic and clinical characteristics between LBC group and non-LBC group.

Variables	LBC group (N = 561)	Non-LBC group (N = 268)	z/χ2	*P*
Age	13(11,14)	12(10,14)	-3.642	<0.001^#^
Sex			0.435	0.509
Male, n (%)	267(47.59%)	121(45.15%)		
Female, n (%)	294(52.41%)	147(54.85%)		
Nation			55.376	<0.001^#^
Han, n (%)	335(59.71%)	86(32.09%)		
Minorities, n (%)	226(40.29%)	182(67.91%)		
Anxiety symptoms, n (%)	248(44.21%)	61(22.75%)	-4.356	<0.001^#^
Total score	23(15,33)	17(11,25)	-6.881	<0.001^#^
Panic/somatic symptoms	5(3,8)	3(2,6)	-5.190	<0.001^#^
Generalized anxiety	5(3,8)	3(2,6)	-6.039	<0.001^#^
Separation anxiety disorder	5(2,7)	3(2,5)	-5.561	<0.001^#^
Social anxiety disorder	6(4,8)	5(3,7)	-3.411	<0.001^#^
School phobia	2(1,3)	1(0,2)	-6.761	<0.001^#^

LBC, left-behind children; #, significant after bonferroni correction.

### 3.2 Socio-demographic and left-behind characteristics by AS status in the LBC group

**Table 2** displays noticeable variations between the AS and non-AS subgroups regarding sex in the LBC group (P < 0.001) and attitude toward parental migration (P = 0.003). Among these factors, the AS subgroup exhibited a greater proportion of females and a decreased prevalence of endorsing supportive attitudes towards their parent(s) migration.

**Table 2:** Comparison of socio-demographic and left-behind features of AS and non-AS subgroups in the LBC group.

Variables	Anxiety symptoms subgroup (N = 248)	Non-Anxiety symptoms subgroup (N = 313)	z/χ2	*P*
Age(years), Median (IQR)	13(11,14)	13(11,14)	-1.932	0.053
Sex			15.371	<0.001^#^
Male, n (%)	95(38.31%)	172(54.95%)		
Female, n (%)	153(61.69%)	141(45.05%)		
Nation			1.115	0.291
Han, n (%)	142(57.26%)	193(61.66%)		
Minorities, n (%)	106(42.74%)	120(38.34%)		
Only child status			5.198	0.023
Yes, n (%)	19(7.66%)	43(13.74%)		
No, n (%)	229(92.34%)	270(86.26%)		
Parental migration status			3.080	0.079
One parent, n (%)	45(18.15%)	76(24.28%)		
Both parents, n (%)	203(81.85%)	237(75.72%)		
Duration of parental migration			2.304	0.316
Less than 1 year, n (%)	118(47.58%)	162(51.76%)		
1–2 years, n (%)	57(22.98%)	56(17.89%)		
More than 2years, n (%)	73(29.44%)	95(30.35%)		
Frequency of communication with migrant parent(s)			3.289	0.193
Weekly, n (%)	191(77.02%)	259(82.74%)		
Monthly, n (%)	32(12.90%)	27(8.63%)		
More than 1 month, n (%)	25(10.08%)	27(8.63%)		
Presence of favorite animal			0.157	0.692
Yes, n (%)	216(87.10%)	269(85.94%)		
No, n (%)	32(12.90%)	44(14.06%)		
Presence of favorite plant			4.535	0.033
Yes, n (%)	189(76.21%)	213(68.05%)		
No, n (%)	59(23.79%)	100(31.95%)		
Availability of confidant			0.882	0.348
Yes, n (%)	227(91.53%)	293(93.61%)		
No, n (%)	21(8.47%)	20(6.39%)		
Availability of help-seeking Figure			3.744	0.053
Yes, n (%)	211(85.08%)	283(90.42%)		
No, n (%)	37(14.92%)	30(9.58%)		
Attitudes towards parental migration			11.647	0.003^#^
Support, n (%)	100(40.32%)	157(50.16%)		
Neutrality, n (%)	80(32.26%)	106(33.87%)		
Opposition, n (%)	68(27.42%)	50(15.97%)		

LBC, left-behind children; AS, anxiety symptoms; #, significant after bonferroni correction.

### 3.3 Risk factors for AS in the LBC group

**Figure 2:**
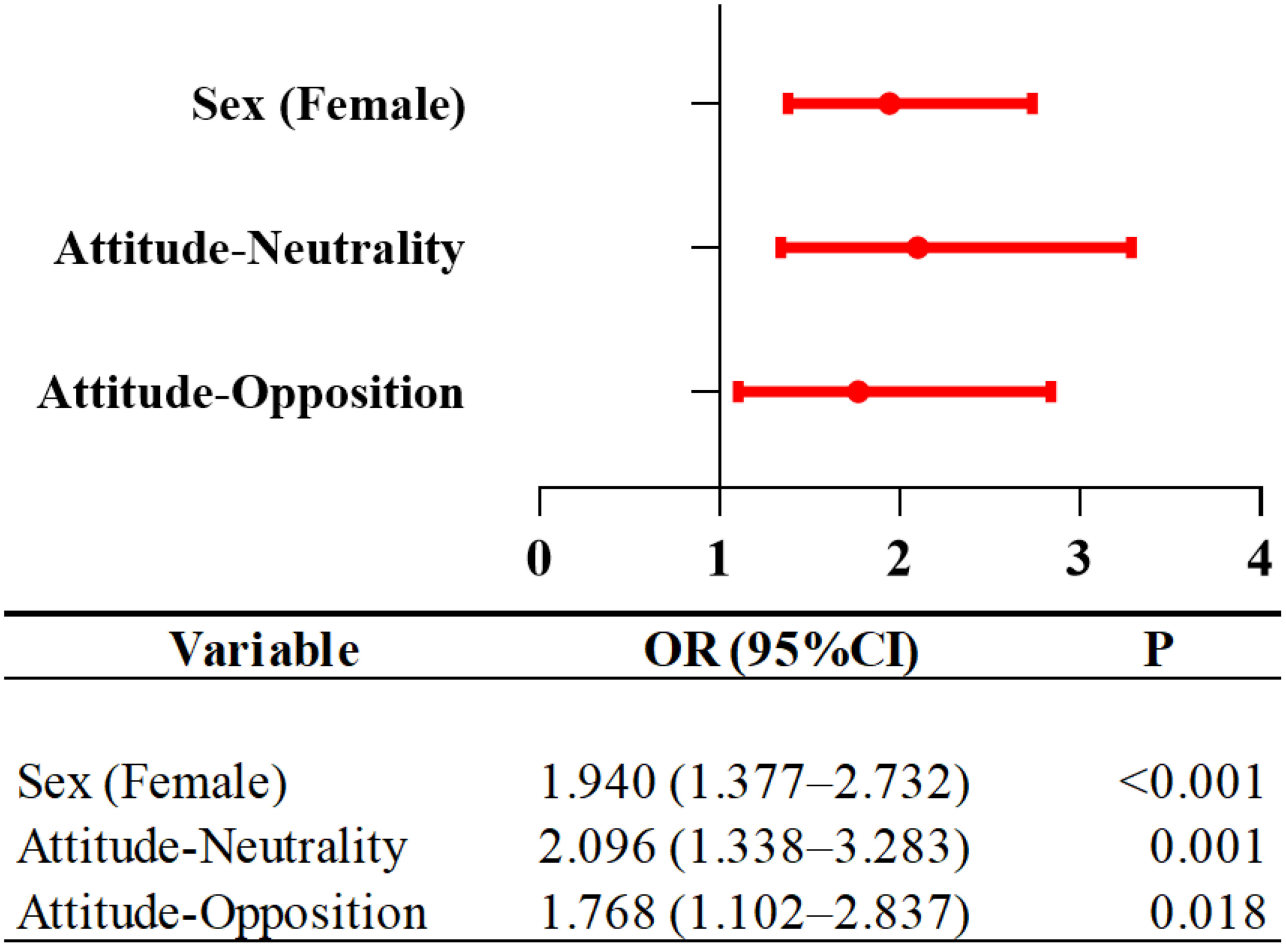
Risk factors associated with anxiety symptoms in LBC. LBC, Left-Behind Children; OR, Odds ratio; CI, Confidence interval.

Our study examined the risk factors of AS in the LBC group. After Bonferroni correction, sex and attitudes toward parental migration remained statistically significant in univariate analysis were therefore included in the multivariable logistic regression model. The analysis revealed that female was 1.940 times more likely to be associated with AS than male in the LBC group (B = 0.663, P < 0.001, OR = 1.940, 95%CI=1.377-2.732). The children with an Attitude-Neutrality had 2.096 times higher odds of AS (B = 0.740, P = 0.001, OR = 2.096, 95%CI=1.338-3.283) compared to Attitude-Support (**Table 3**, **Figure 2**).

**Table 3:** Factors associated with anxiety symptoms in LBC.

Items	B	Waldstatistic	*P*	OR	95%CI
Sex					
Male	–	–	*-*	1.000	–
Female	0.663	14.370	<0.001	1.940	1.377-2.732
Attitude					
Support	–	–	–	1.000	–
Neutrality	0.740	10.443	0.001	2.096	1.338-3.283
Opposition	0.570	5.588	0.018	1.768	1.102-2.837

LBC, left-behind children; OR, Odds ratio; CI, Confidence interval. #, significant after bonferroni correction.

### 3.4 Associations between SCARED scores and clinical features

**Figure 3:**
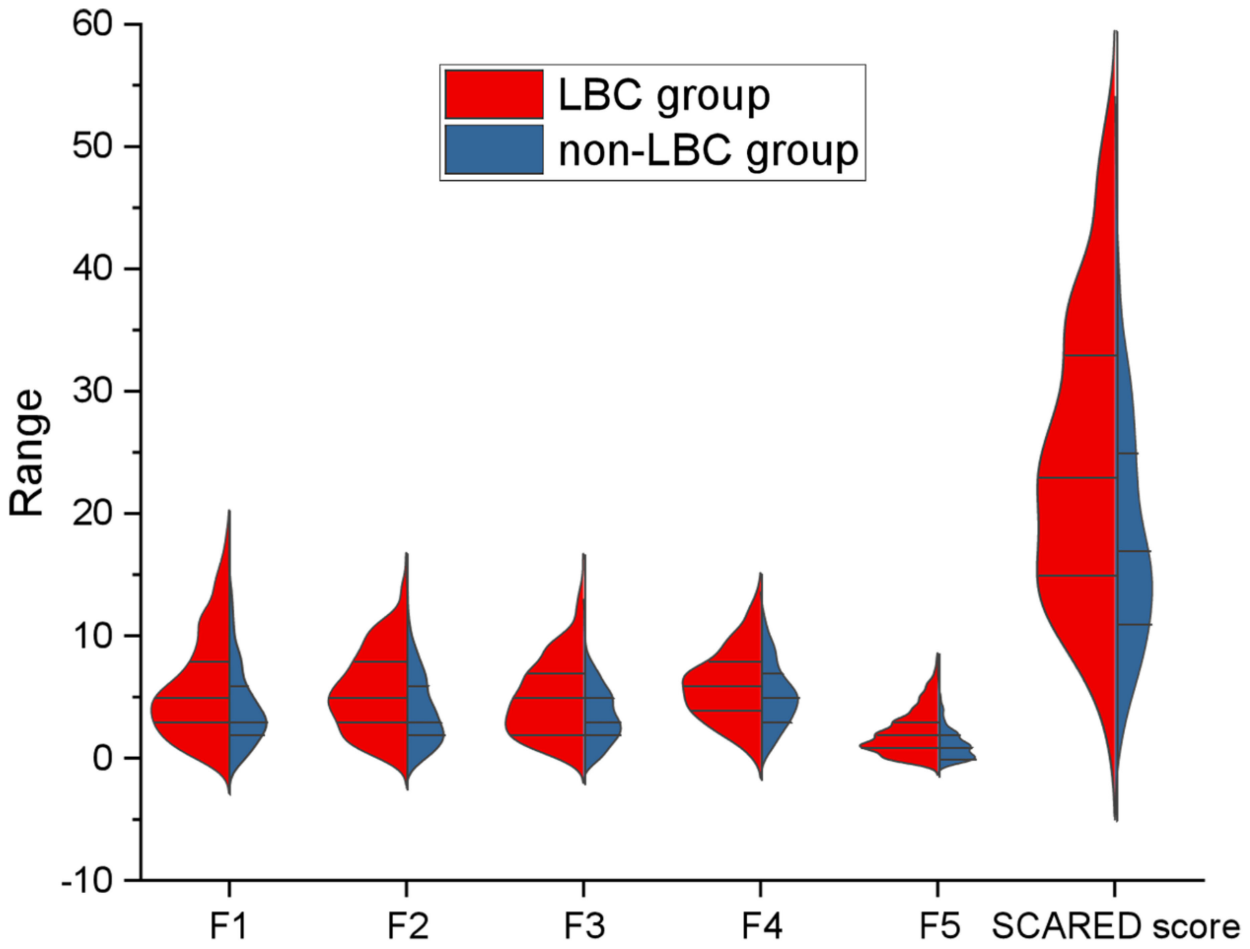
SCARED Scores Classification of the LBC and Non-LBC Groups. LBC, Left-Behind Children.

As shown in **Figure 3**, the violin plots of the SCARED subscales and total scores demonstrated that LBC consistently exhibited higher scores across all dimensions, including panic/somatic symptoms, generalized anxiety, separation anxiety, social phobia, and school phobia, compared with non-LBC. The differences in total scores were particularly pronounced.

As presented in **Table 4**, the multivariable linear regression analysis included all socio-demographic characteristics and left-behind features, and identified several variables that were significantly associated with SCARED scores. Sex was significant, with females showing higher SCARED scores than males (B = 4.127, β = 0.178, t = 4.106, P < 0.001). Frequency of communication with migrant parent(s) was also associated with SCARED scores (B = 1.996, β = 0.105, t = 2.580, P = 0.010), with children communicating monthly or at longer intervals reporting higher SCARED scores compared with those communicating weekly. In addition, Attitudes towards parental migration were significant (B = 1.880, β = 0.127, t = 3.086, P = 0.002), with neutral and opposed attitudes linked to higher SCARED scores than supportive attitudes. Lastly, Availability of help-seeking figure (B = 3.020, β = 0.085, t = 2.038, P = 0.042) indicated that children without someone to seek help from had higher SCARED scores compared with those who did. These variables were independently associated with SCARED scores.

**Table 4:** Multivariate linear regression analysis of factors associated with SCARED scores.

Variable	B	SE	Beta	t	p-value
Sex	4.127	1.005	0.178	4.106	0.000
Age	0.391	0.215	0.077	1.816	0.070
Ethnicity	1.278	0.984	0.054	1.299	0.195
Only child status	1.986	1.536	0.054	1.293	0.197
Parental migration status	0.886	0.657	0.045	1.349	0.178
Duration of parental migration	0.459	0.573	0.035	0.802	0.423
Frequency of communication with migrant parent(s)	1.996	0.774	0.105	2.580	0.010
Attitudes towards parental migration	1.880	0.609	0.127	3.086	0.002
Availability of confidant	0.491	0.819	0.019	0.600	0.548
Availability of help-seeking figure	3.020	1.482	0.085	2.038	0.042
Presence of favorite animal	0.331	1.472	0.010	0.225	0.822
Presence of favorite plant	-1.854	-1.192	-0.072	-1.555	0.121

## 4 Discussion

This study is the first to identify the occurrence and associated elements and perspectives of the migration of parent(s), particularly of AS, in a vast dataset of LBC in the southern region of Yunnan Province, a region populated by ethnic minorities. Our results revealed a 2.285-fold increase in AS prevalence in the LBC cohort (44.21%) compared to the non-LBC cohort (22.75%). Furthermore, “Sex-Female”, “Attitude-Neutrality”, and “Attitude-Opposition” were associated with AS in LBC independently. Moreover, “Sex-Female”, “Frequency of communication with migrant parent(s) monthly and more than one month”, “Attitude-Neutrality and Attitude-Opposition towards migration of parent(s)”, and “children did not have someone for help” linked to the SCARED score in LBC group.

In this study, the proportion of AS in the LBC group was 2.285 times higher than in the non-LBC group. Previous studies reported similar findings. For instance, a meta-analysis found that the prevalence of severe mental health issues among LBC is almost 2.7 times greater than in non-LBC individuals. The primary mental health challenges encountered by LBC are anxiety disorders (19). Another meta-analysis, with a sample of 264,967 children, revealed that children who were left-behind had a 1.85 times higher risk of developing anxiety than those whose parents did not migrate (1). Furthermore, a study in rural areas of Chongqing, Guizhou, and Anhui provinces in China discovered that children who experienced parental separation at a young age had increased AS (20). However, previous research has yielded conflicting results. A study examining mental health status and influencing factors in LBC found no statistically significant variance in mental health challenges between LBC and non-LBC individuals (7). Another study showed that LBC did not have worse mental health than their control counterparts (21). Additionally, a cross-sectional study, conducted in North China, with a sample of 2283, illustrated that children parents’ migration was not associated with anxiety in children (8).

The discrepancies observed may stem from two main causes. Firstly, the diversity of study participants can impact AS in LBC, with age being a significant factor. Tao et al. found that the LBC ranged from three to five years old (21). Similarly, Zhang et al.’s study involved 4,187 children aged 3–16 years (7). Some studies included children aged 10–17 (20) and 10–18 years (8). Furthermore, Fellmeth et al. conducted a mate analysis covering 0–19 years (1). The age differences between the subjects could explain the disparities in the results. Our study’s sample of ages ranging from 7 to 17 years can better represent the situation of school-age children and adolescents. Secondly, the economic conditions may explain the differences in the results. A study showed that LBC had a caregiver with better economic conditions, which was associated with differences in emotional status (21). Furthermore, another study showed that low-income populations may experience higher frequencies of anxiety-related symptoms (22). Madasu et al. demonstrated that individuals from lower- and middle-income backgrounds are more likely to experience anxiety disorders (23). Another study provided interdisciplinary proof and insight into the reciprocal cause-and-effect connection between poverty and anxiety (24). Therefore, variations in the age groups of the participants and economic circumstances could potentially explain these discrepancies.

This study indicated that the sex of female was a risk factor associated with AS in LBC, consistent with previous studies. A study revealed that females have twice the risk of developing anxiety than males (25). An earlier study declared that the female sex was associated with the presence of anxiety disorder (23). The epidemiological sex difference in anxiety disorder has good characteristics. Females are at a heightened risk of experiencing anxiety, possibly due to the following three factors. Firstly, hormones such as estradiol and progesterone could potentially contribute to sex-based differences in anxiety by 1) increasing susceptibility to factors linked to developing anxiety disorders and 2) aiding in the persistence of AS post-development (25). Furthermore, another study also showed that women are more likely to experience anxiety during times of hormonal flux (26). Secondly, a study presented evidence indicating that trait anxiety correlated with increased blood flow in a cluster of areas comprising the amygdala, anterior insula, and fusiform cortex. Furthermore, variations in the progression of cerebral blood flow during adolescence could serve as a crucial factor in emotional neuroscience that underlies sex disparities in anxiety and mood disorders (27). Thirdly, an animal experimental study suggested that sex differences in the configuration of ϒ-amino butyric acid (GABA) GABAergic interneurons in the cortico-amygdala-hippocampal network control anxiety (28).

A recent study has examined the psychological consequences of parental migration among LBC, including associations with anxiety-related outcomes (29). Building on this emerging evidence, this study examined the association between LBC’s attitudes toward parental migration and AS. The study revealed that the likelihood of AS was 2.096 times greater in individuals with a neutral attitude than in those with a supportive attitude and 1.768 times higher in individuals with an opposition attitude. There are two possible explanations for this result. Firstly, LBC’s attitude towards parents’ migrant work may be influenced by LBC’s interpretation of the meaning of parents’ migrant work. LBC, who is neutral or opposed to parents’ going out to work, may not understand the meaning of parents’ going out to work, which may be a reason for LBC’s anxiety. A previous study illustrated that numerous young individuals endure parental migration as a manifestation of abandonment or rejection, resulting in emotional displacement and potentially displaying indicators of disrupted behaviours (30). Additionally, extensive research has been conducted on the correlation between anxiety and meaning. A study showed that anxiety correlated positively with searching for meaning and negatively with the presence of meaning (31). Separate research on the significance of life validated that anxiety diminished with heightened levels of meaning, in contrast to average and low levels of meaning (32). Furthermore, a study on cognitive behavioural therapy intervention illustrated that meaning-making is a mediator of anxiety reduction in participants (33). In our study, LBCs who were neutral and opposed may not have found the meaning of their parents going out to work, causing anxiety.

Secondly, according to attachment theory, as a way to explain human bonding, different attachment styles have different responses to stress, such as parental migration. Attachment security corresponded to low anxiety scores and positive views of the self and others. Previous studies uncovered that securely attached participants had the lowest scores for anxiety and the highest scores on the resiliency measure (34), primed to protect against relapse of fear (35). Children with secure attachment may provide support to their parents who are migrant workers. Conversely, insecure attachment could be identified as a significant risk factor for the onset of AS. For instance, previous studies have found that insecure attachment is a risk factor for anxiety symptomatology (36) and can predict prospective symptoms of anxiety (37). Meta-analyses results showed that insecure attachment was significantly associated with anxiety (38). In our study, LBC with neutral and opposed attitudes may have insecure attachments to their parents. Individuals with insecure attachment have increased biased attention toward emotional stimuli, especially under stress (39). Additionally, people with a vulnerable attachment style encounter tension tend to exhibit intense emotional reactions (40) and have a higher chance of struggling to manage their feelings and engage effectively with peers, which could exacerbate anxiety and is linked more strongly to coping and challenges during less intense conflict situations (41). Therefore, children with insecure attachments cannot offer support to their parents, who are migrant laborers. Consequently, when assisting LBC, it is crucial to focus on fostering beneficial attachment experiences, as these could favor the formation of children’s enduring attachment characteristics (42).

Future studies should adopt longitudinal designs to clarify the temporal and causal relationships between parental migration-related factors and AS among left-behind children. In addition, incorporating objective socioeconomic indicators and contextual variables, such as caregiver mental health and family functioning, would provide a more comprehensive understanding of the mechanisms underlying anxiety in this population. Further research conducted in diverse regions and cultural settings is also warranted to enhance the generalizability of these findings and to inform targeted interventions for left-behind children.

## 5 Limitation

This study has several limitations. Firstly, being a cross-sectional survey, the findings are limited to showing correlations rather than causations. Future studies should use a longitudinal design to investigate and analyze the causal relationship between mental health and various influencing factors. Secondly, the current study paid insufficient attention to understanding the mechanisms by which specific aspects of being left behind impact the mental health of LBC. Therefore, further research must identify the key characteristics contributing to this impact on LBC. Thirdly, the reliance on self-reported measures may introduce response biases, and the absence of contextual variables, such as caregiver mental health limits a more comprehensive understanding of influencing factors. Fourthly, socioeconomic indicators, such as household income, parental education, and caregiver employment status, were not directly measured in this study, which may have limited our ability to fully account for socioeconomic confounding. Lastly, the study sample consisted solely of participants from one province in southern China. Consequently, caution should be exercised when generalizing these findings to the nation.

## 6 Conclusion

Our study suggests that the prevalence of AS was 44.21% in the LBC group than in the non-LBC group, representing a 2.285-time increase. Factors independently linked to AS in LBC were “Sex-Female”, “Attitude-Neutrality”, and “Attitude-Opposition”. Consequently, we recommend an increased focus on LBC, specifically among female children with opposed attitudes toward parental migration. Longitudinal studies should be conducted to confirm this conclusion.

## Data Availability

The raw data supporting the conclusions of this article will be made available by the authors, without undue reservation.
